# Introduction to *RSC Advances* themed collection on Nanoarchitectonics Advances: Bridge over Nanotechnology and Materials Science

**DOI:** 10.1039/d4ra90076f

**Published:** 2024-08-02

**Authors:** Katsuhiko Ariga, Hiromitsu Maeda, Stéphane A. Baudron, Yulan Chen

**Affiliations:** a National Institute for Materials Science & The University of Tokyo Japan; b Ritsumeikan University Japan; c Université de Strasbourg, CNRS, CMC UMR 7140 4 rue Blaise Pascal F-67000 Strasbourg France; d Jilin University China

## Abstract

A passport to a method for everything in materials science.
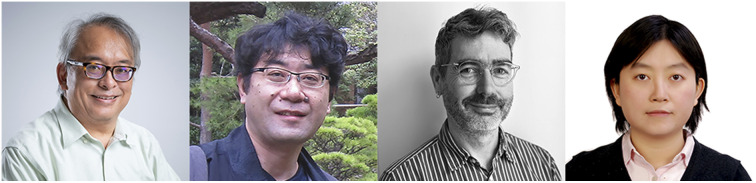

The history of human development has depended on the discovery and development of new substances. In particular, the creation of new functional materials has enhanced the richness of human life. In ancient times, humans extracted materials from nature and processed them to make tools. In the 20th century, the science of synthesizing materials was established, including organic chemistry, inorganic chemistry, polymer chemistry, coordination chemistry, supramolecular chemistry, and biochemistry, as well as a range of material sciences encompassing all of these. Mankind is now able to fabricate materials based on rational science. Furthermore, the influence of a nanostructure on function has become clear. The functionality of the same substance can change significantly if its internal structure differs. The importance of nanostructure has been demonstrated through nanotechnology. In addition, nanoarchitectonics has been proposed as a new concept that expands the field of nanotechnology and takes it even further.

Just as Richard Feynman founded nanotechnology in the mid-20th century, nanoarchitectonics was proposed by Masakazu Aono at the beginning of the 21st century as a post-nanotechnology concept. Nanotechnology can be used to observe and manipulate atomic, molecular, and nano-sized structures to reveal their specific properties. It is necessary to assemble materials based on nano knowledge and information. This is what nanoarchitectonics is all about. The goal of nanoarchitectonics is to construct materials with precise structures from nanoscale units, such as atoms, molecules, and nanomaterials, to realize high performance. Beyond well-known self-assembly and related strategies, nanoarchitectonics aims to build material structures from multiple building blocks, including more asymmetric and hierarchical motifs. Nanoarchitectonics bridges the missing link between nanotechnology and materials science.

The philosophy of nanoarchitectonics is to build functional materials and systems by organizing nano-units into material structures. The properties of the resulting nanostructure may differ from the properties of the original nano-units, resulting in the creation of new functionality through their internal synergistic interactions. That unexpected functionality may be contained in a very large number of nano-units, either by self-assembly or by deliberate organization.

New theoretical and computational methods are envisioned to facilitate the manufacturing process of nanoarchitectonics. In particular, recent developments in artificial intelligence may further expand this potential. The integration of nanoarchitectonics with material informatics has also been proposed.

The impact of nanoarchitectonics extends beyond the fabrication of such structures to the development of nanostructured materials useful for sensing, catalysis, energy, environmental, and biomedical applications. According to this background, this themed issue collects exciting research papers that discuss the construction of functional material systems based on nanoarchitectonics. The aspects are diverse. Tahara and coworkers report, for example, on the effect of the core size on the structure and chirality of self-assembled molecular networks using two aromatic carboxylic acid derivatives with frameworks displaying *C*_3h_ symmetry (https://doi.org/10.1039/D3RA05762C). This is a fundamental exploration of the evolution of structure at the molecular level. Minami and coworkers show an example of precise sensing of metal ions by nanoarchitectonic polythiophene structures printed on papers (https://doi.org/10.1039/D3RA08429A). Akiyama and coworkers investigate photoelectric conversion properties using nanoarchitectonically fabricated films of fullerene derivatives and polythiophene (https://doi.org/10.1039/D3RA05150A), which is a key to the development of optoelectronic devices. Li, Wang and co-workers demonstrate the use of nanoarchitectonics of porous polymers for the alleviation of doxorubicin-induced cardiotoxicity *via* passive targeted release (https://doi.org/10.1039/D2RA07410A).

Thus, even if we limit ourselves to the examples in the papers in this themed issue, diverse applications of nanoarchitectonics emerge. All matter is fundamentally composed of atoms and molecules. Therefore, nanoarchitectonics is a methodology that may apply to all material synthesis. In ancient times, humans extracted materials from nature and processed them based on experience. We have now arrived at the concept of artificially creating all materials. Nanoarchitectonics can be a passport to the method for everything.

